# Three-dimensional fluid–structure interaction modelling of the venous valve using immersed boundary/finite element method

**DOI:** 10.1016/j.compbiomed.2024.109450

**Published:** 2025-02

**Authors:** Bo Wang, Liuyang Feng, Lei Xu, Hao Gao, Xiaoyu Luo, Nan Qi

**Affiliations:** a Research Center for Mathematics and Interdisciplinary Sciences, Shandong University, Qingdao, 266237, China; b School of Mathematics and Statistics, University of Glasgow, Glasgow, G12 8QQ, UK; c Shandong Provincial Hospital Affiliated to Shandong First Medical University, Jinan, 250021, China; d Frontiers Science Center for Nonlinear Expectations, Ministry of Education, Qingdao, 266237, China

**Keywords:** Fluid–structure interaction, Venous valve, Immersed-boundary finite element method, Three-dimensional framework

## Abstract

Research on venous hemodynamics is pivotal for unravelling venous diseases, including varicose veins and deep vein thrombosis, essential for clinical management, treatment and artificial valve design. In this study, a three-dimensional (3D) numerical simulation, employing the immersed boundary/finite element method, is constructed to explore the fluid–structure interaction (FSI) between intravenous blood and venous valves. A hyperelastic constitutive model is used to capture the incompressible, nonlinear mechanical response. Our findings reveal the periodic characteristics of valve movement and intravenous blood flow throughout the cardiac cycle, alongside quantified physiological parameters such as blood pressure, flow rate, geometric orifice area, and stress–strain distribution on venous valve surfaces. The study unveils a significant correlation between dynamic valve motion and vortices within the venous sinus. Stress and strain concentrate primarily at the free edge of venous valves, which is in contrast to 2D modelling. Moreover, increased hydrostatic venous pressure is found to be the key to venous vessel dilation. The effects of fibrosis and atrophy of venous valves on venous hemodynamics are compared and analysed. This FSI numerical study introduces a fully 3D framework for modelling the venous system, expected to provide crucial references for understanding the development and mechanism underlying venous diseases, thereby furnishing a scientific underpinning for their prevention, diagnosis, and treatment.

## 1 Introduction

Veins play a critical role in the cardiovascular system, storing 85% of the body’s blood and facilitating the return of deoxygenated blood to the heart. Reduced venous return and prolonged blood transit times from the lower extremities can have a systemic effect that mimics heart failure [1]. Every year, nearly 10 million people worldwide suffer from thromboembolism [2]. In America alone, venous disease affects over 80 million people, leading to a range of clinical manifestations including varicose veins, oedema, stasis dermatitis, and even venous ulcers. Understanding biomechanical characteristics, such as venous reflux and venous wall dilation, is essential for prevention, control management and treatment of these conditions.

With the advancements in clinical inspection and medical imaging technologies [3], [4], our comprehension of venous systems is rapidly evolving. However, these technologies commonly focus on static, instantaneous biomechanical representations, making it challenging to quantify tissue changes in the venous valve system and their time-dependent response to dynamic mechanical stimuli. Moreover, the pathology of venous diseases is closely linked to the hemodynamics of the entire venous system, the biological properties of venous tissues, and the collaboration among all venous components. Exploring appropriate mathematical models and numerical methods to analyse fluid–structure interaction (FSI) between venous tissue and blood in veins can significantly enhance our knowledge of the pathological mechanism of venous diseases and aid in the development of new and effective surgical treatment strategies.

Despite the proven effectiveness of numerical methods in solving complex biomechanical problems and their widespread use in heart and artery research [5], [6], [7], [8]-providing insights that are often difficult or impossible to obtain through experimental methods alone-there has been relatively limited focus on numerical studies of the venous system and its associated diseases. From the perspective of pure fluid dynamics, Packer et al. used computational fluid dynamics (CFD) analysis to validate the effectiveness of one type of artificial valve [9]. Wang et al. employed CFD simulations to model the hemodynamic changes in venous diseases caused by stent implantation [10]. Most studies, however, focused on the full interaction between fluid and solid. For instance, Zervides et al. established a one-dimensional collapsible tube model to quantify the pressure shielding effect of venous valves and the changes in various system parameters [11]. Wijeratne et al. developed a two-dimensional model to simulate flow dynamics around the human vein and venous valves, visualising the process of valve opening and vortex formation [12]. Liu et al. utilised an improved immersed boundary method to analyse the influence of venous valves with different lesions on blood flow properties, offering a prospective understanding of the effect of valve lesions on the venous valve cycle [13]. Soifer et al.’s work revealed the impact of diseased valves on adjacent valves [14]. Buxton et al. used the lattice Boltzmann method to characterise the motion of linear venous valves [15]. Tien et al.’s work guided artificial venous valve design through FSI simulation [16]. Recently, Lin et al. investigated the mechanical changes in venous diseases induced by drug therapy, providing a precise analysis of critical clinical issues in venous thrombosis [17]. While numerical simulation can provide invaluable information that is difficult to measure experimentally, aiding in a deeper understanding of veins and assisting in the treatment of venous insufficiency, numerical modelling of veins is still in its infancy. Two-dimensional (2D) geometrical models and linear mechanical properties of venous tissue are often oversimplified and challenging to replicate in realistic biological and pathological scenarios.

The key contribution of this paper lies in the development of a fully three-dimensional (3D) framework of veins in the lower extremity, focusing on the fluid–structure dynamics of venous valves. The 3D model incorporates hyperelastic and nonlinear material properties, enabling complicated clinically relevant studies that are not possible with 2D models. This marks a major advancement, as it allows for the analysis of complex physiological behaviours, including stress–strain distributions, flow rate variations, and intravascular vortex formation-all critical for understanding venous disease progression. In particular, the immersed boundary method, using a finite element description [18], [19], is elaborated in Section 2, and nonlinear elasticity material models are employed primarily according to data in the 2D model in [13] for comparison. Section 3 presents simulation results, encompassing mechanical and hydrodynamic information inside the vein and surrounding the venous valve. The discussion with regard to diseased venous modelling and conclusion are subsequently presented in the last two sections.

## 2 Methods

### 2.1 Immersed boundary (IB) method

The immersed boundary(IB) method is a mathematical modelling approach employed to solve the fluid–structure interaction(FSI) problems involving an elastic structure immersed in a viscous incompressible fluid [19]. This method utilises a fixed Eulerian framework to represent the motion within the flow field and a moving Lagrangian framework to capture the deformation of immersed elastic structures. All fluid properties are computed at stationary points in space that do not move with the flow. The interaction between the fluid and the immersed structure is handled through interpolation and force spreading between the Lagrangian points of the structure and the Eulerian fluid grid.

**Fig. 1:**
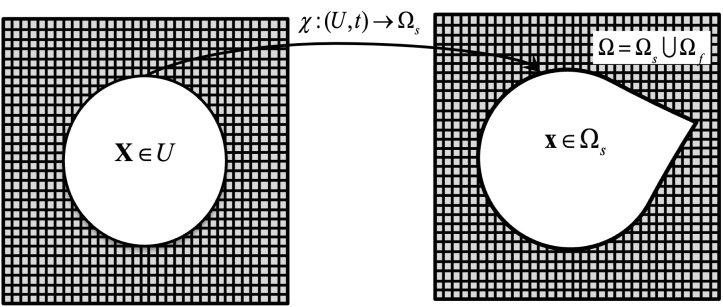
Evolution of a continuum material in the IB method.

As depicted in Fig. 1, let $x\in \Omega \subset R^{3}$ denote the fixed physical coordinates, or the Eulerian coordinates, and $X\in U\subset R^{3}$ denote the Lagrangian reference coordinates. The mapping between these two frameworks is described by $\chi :\left(U,t\right)\mapsto \Omega_{s}$ representing the physical trajectory of material point X at time t. Thus, $x=\chi \left(X,t\right)\in \Omega_{s}$ denotes the physical region occupied by the immersed structure, while the physical region occupied by the fluid at time t is given by $\Omega_{f}=\Omega \setminus \Omega_{s}$.

The governing equations of motion for the FSI system can be expressed as:

(1) $$\rho \left(\frac{\partial u}{\partial t}\left(x,t\right)+u\left(x,t\right)\cdot \nabla u\left(x,t\right)\right)=-\nabla p\left(x,t\right)+\mu \nabla^{2}u\left(x,t\right)+f\left(x,t\right),$$

(2) ∇⋅u(x,t)=0,

(3) $$f\left(x,t\right)=\int_{U}F\left(X,t\right)\delta \left(x-\chi \left(X,t\right)\right)dX,$$

(4) $$\int_{U}F\left(X,t\right)\cdot V\left(X\right)dX=-\int_{U}P\left(X,t\right):\nabla_{X}V\left(X\right)dX,$$

(5) $$\frac{\partial \chi }{\partial t}\left(X,t\right)=\int_{\Omega }u\left(X,t\right)\delta \left(x-\chi \left(X,t\right)\right)dx$$

Eqs. (1), (2) represent the incompressible Navier–Stokes equations, where ρ denotes fluid density, μ denotes dynamic viscosity, u(x,t) represents the Eulerian velocity field, and p(x,t) represents the Eulerian pressure field. F(X,t) and f(x,t) are the Eulerian and Lagrangian forms of the elastic force density associated with the deformation of the venous tissues, respectively. The numerical framework employed in this work is the finite element (FE) version of the immersed boundary (IB) method or the IB/FE method [18]. This approach not only mitigates the requirement for the structural Lagrangian mesh to be finer than the fluid’s Eulerian grid, but also enables the representation of the elasticity of the solid structure with FE discretisation. Thus, the tensor P(X,t) is introduced to represent the first Piola–Kirchhoff elastic stress tensor corresponding to the Lagrangian material coordinates, while V(X) denotes an arbitrary Lagrangian test function in the FE formulation. The three-dimensional Dirac delta function δ(x) implements the force spreading in Eq. (3) and velocity-restriction in Eq. (5) between the Eulerian and Lagrangian coordinate systems. More specifically, the evolution of χ(X,t) over time is interpolated from the fluid velocity field on the Eulerian grid to the Lagrangian points. The forces generated by the motion of the immersed structures are computed at the Lagrangian points and then spread back to the Eulerian grid using the same δ function (or its smoothed approximation). The fluid density ρ and dynamic viscosity μ are assumed to be uniform in our work.

The spatial discretisation of the incompressible Navier–Stokes equations is carried out using a staggered-grid finite difference scheme, while the elastic materials are discretised using a finite element interpolation scheme. The components of the Eulerian velocity field u and body force f are approximated at the edge centres of the Cartesian grid cells; however, the pressure p is approximated at the cell centres. Time discretisation is performed using the Runge–Kutta method.

### 2.2 Material model

A nonlinear hyperelastic constitutive model based on the formation proposed by [20], [21] is used to describe the venous wall and valves. The sinus is assumed to have the same material property as the wall. Materials are all assumed to be incompressible and are expressed as:

(6) $$\Psi^{\text{wall}}=c_{0}\left(I_{1}-3\right)+c_{1}{\left(I_{1}-3\right)}^{2}+\frac{\beta }{4}{\text{log}}^{2}\left(I_{3}\right),$$

(7) $$\Psi^{\text{valve}}=c_{2}\left(\exp\left[c_{3}{\left(I_{1}-3\right)}^{2}\right]-1\right)+\frac{\beta }{4}{\text{log}}^{2}\left(I_{3}\right),$$

in which $I_{1}=\text{tr}\left(F^{T}F\right)$ represents the principal invariant in terms of the deformation gradient tensor F, defined as $F=\frac{\partial \chi }{\partial X}\left(X,t\right)$. The term $\frac{\beta }{4}{\text{log}}^{2}\left(I_{3}\right)$ is a volumetric energy that acts to penalise compressible deformations, in which $I_{3}=\text{det}\left(F^{T}F\right)$ and β is chosen to be 500kPa. Here, the fibre-reinforced terms are omitted, similar to [13] for further comparison. The material coefficients $c_{i}$, where i=1,2,3, are summarised in Table 1, again following the data in [13]. The relationship between the first Piola–Kirchhoff stress tensor and the strain energy function can be expressed as $P\left(X,t\right)=\frac{\partial \Psi }{\partial F}\left(X,t\right)$ so that

(8) $$P^{\text{wall}}=2c_{0}F+4c_{1}\left(I_{1}-3\right)F\underline{-2c_{0}F^{-T}-4c_{1}\left(I_{1}-3\right)F^{-T}}+\beta \text{log}\left(I_{3}\right)F^{-T},$$

(9) $$P^{\text{valve}}=4c_{2}c_{3}\left(I_{1}-3\right)\exp\left[c_{3}{\left(I_{1}-3\right)}^{2}\right]F\underline{-4c_{2}c_{3}\left(I_{1}-3\right)\exp\left[c_{3}{\left(I_{1}-3\right)}^{2}\right]F^{-T}}\;+\beta \text{log}\left(I_{3}\right)F^{-T},$$

where the underlined terms are included to ensure P is zero when F is an identity tensor. This modification is found to reduce the magnitude of spurious volume loss caused by the pressure discontinuities across the fluid–structure interface [22].

**Table 1:** Material parameters for different parts in the model [13].

	$c_{0}\;\left[\mathrm{kPa}\right]$	$c_{1}$	$c_{2}\;\left[\mathrm{kPa}\right]$	$c_{3}\;\left[\mathrm{kPa}\right]$
Wall	–	–	2.00	187.5
Valve	417	0.06	–	–
Sinus	–	–	0.50	46.875

### 2.3 Geometry model

**Fig. 2:**
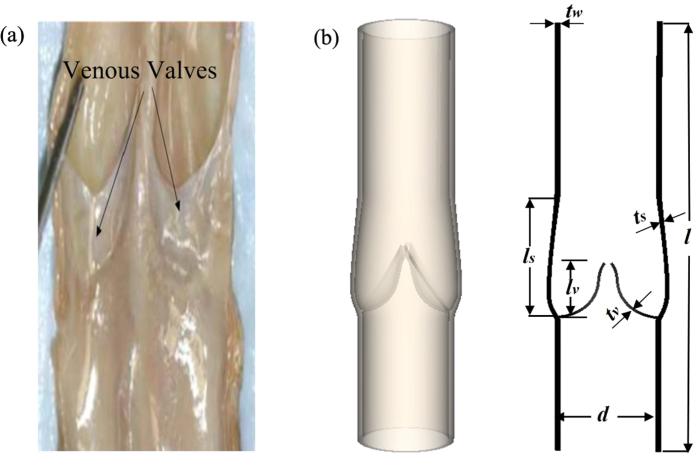
(a) The anatomy of a pair of venous valves exposed from a dissected bovine vein and (b) a 3D venous model with all the typical geometric parameters labelled.

In the numerical simulation, specific geometric parameters of the venous valve are adopted following the study by [13]. The geometry of the vein segment, including the valve sinus and leaflets, is based on dimensional parameters from a typical bovine saphenous vein, resulting in an idealised geometry. Some parameters are scaled similarly to those in [23], [24] and are listed in Table 2. As observed in Fig. 2(a), the cusps of the venous valves are half-moon-shaped [25]. The free edges and the belly region of the cusps are curved in a parabolic shape, which is characteristic of venous valves. The computational vessel geometry is designed using SolidWorks 2022, beginning with a 2D planar sketch derived from these anatomical measurements. The final 3D geometry is created using rotational surface generation and mirroring, as depicted in Fig. 2(b). It is noteworthy that a very small gap exists the two valves to allow for the initialisation of valve opening. Once opened, the valve motion is governed by the momentum equations solved throughout the entire system.

**Table 2:** Geometric parameters for the venous valve model.

Name	Label	Dimension [cm]
Wall length	l	3.200
Wall luminal diameter	d	0.691
Wall thickness	$t_{w}$	0.020
Valve depth	$l_{v}$	0.500
Valve thickness	$t_{v}$	0.020
Sinus depth	$l_{s}$	1.000
Sinus thickness	$t_{s}$	0.015

**Fig. 3:**
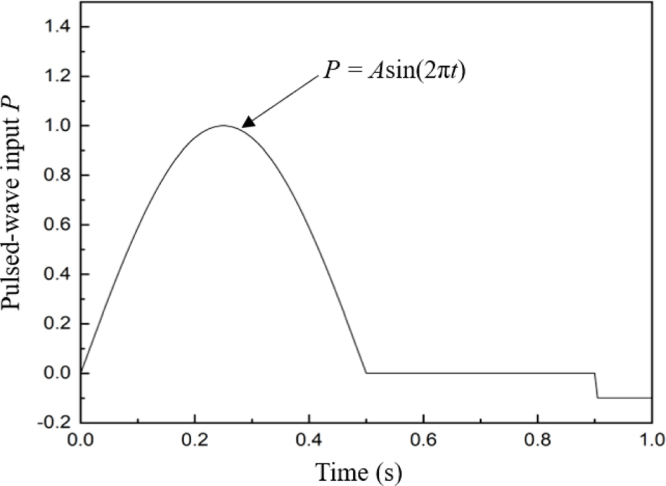
The profile of the pressure difference between the inlet and outlet of the vein in one cardiac cycle, A=0.5mmHg.

**Fig. 4:**
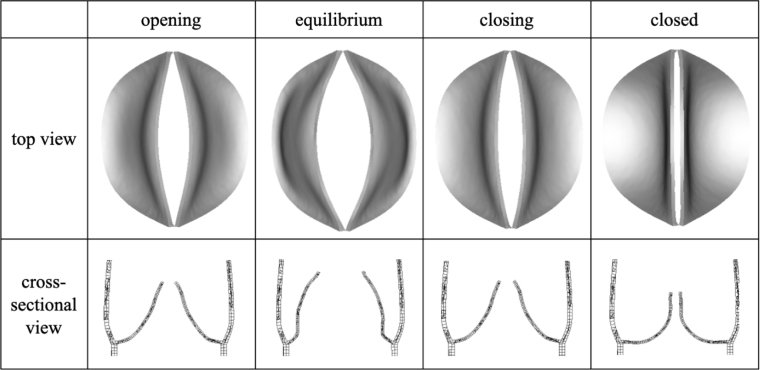
The shapes of the venous valve in opening, equilibrium, closing, and closed phases from both top and cross-sectional views.

**Fig. 5:**
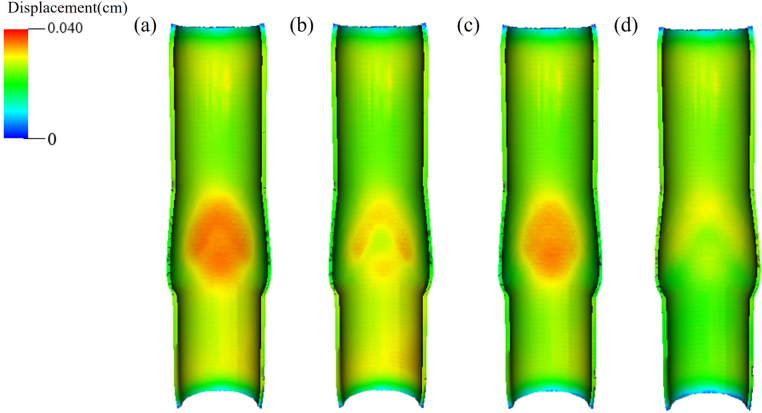
The magnitude of wall displacement in (a) opening, (b) equilibrium, (c) closing, and (d) closed phases.

**Fig. 6:**
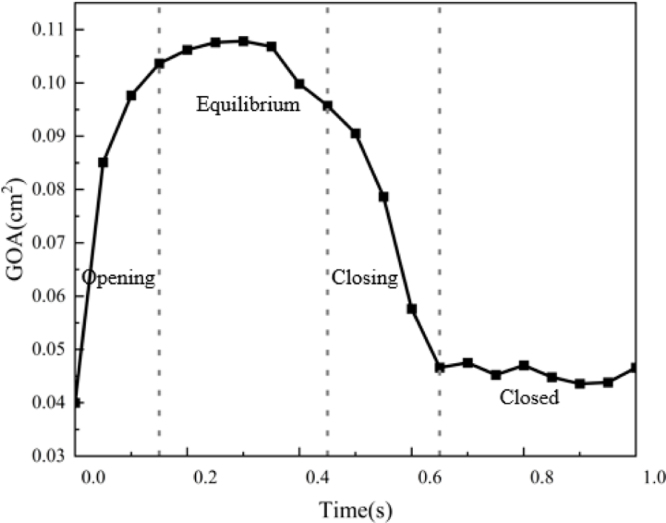
Change in geometric orifice area(GOA) during a whole cycle.

### 2.4 Numerical setup and boundary conditions

In the simulation, the blood is considered a Newtonian fluid with its density ρ of $1.08\;{\mathrm{g/cm}}^{3}$ and dynamic viscosity μ of 0.036Pas. The ends of the vein are all fixed and immersed in a 1cm×1cm×3cm fluid domain. The fluid box is discretised in a Cartesian grid with 50 × 50 × 150 spacings, respectively, which fully accounts for the dimensional differences between venous vessels and valves. A typical simulation comprises 74,294 finite elements and 74,967 nodes. Compatibility of the FE mesh, particularly at the junction between the valves and the sinus, is ensured by sharing a common set of nodes. This guarantees a smooth transition in both geometry and mechanical behaviour at the interface. It is worth noting that the structural mesh is coarser than the fluid mesh, as permitted in the IB method with FE description; detailed information is provided in [18]. The mesh convergence test for the coupled model is conducted with two different grid spacings, 50 × 50 × 150 (which is used in this study) and 100 × 100 × 300, leading to nearly identical results in maximum and average displacements at the equilibrium phase, with a difference of less than 1%.

Pressure boundary conditions are applied on both ends of the vein, where the inlet pressure at the distal end is dynamic, as shown in Fig. 3, where the first half cycle is represented by the sinusoidal function of Asin(2πt). In the last 0.1s during a cycle, a small negative pressure difference between the inlet and outlet was applied to achieve better valve closure. In a supine position, the mean hydrostatic pressure in the lower extremity veins is 4mmHg, and the dynamic pressure drops from 12∼17 mmHg at the end of the capillaries to 4∼7 mmHg at the heart [26]. Hence, in the model, an initial hydrostatic pressure of 4mmHg is applied to both ends, and the maximum pressure difference is set to 1mmHg, taking into account the length of the vein considered herein. Zero-pressure boundary conditions are applied along the remainder of the domain boundaries, similar to our previous study [27].

One typical cardiac cycle of valve motion is 1s [13], and a time step $1\times 10^{-5}\;s$ is chosen in the explicit time-stepping scheme to ensure numerical convergence after computational experimentation, which is highly correlated to the Courant–Friedrichs–Lewy condition when solving partial differential equations numerically. This choice is comparable to time-stepping schemes used in other studies on heart valve FSI problems [5], [28], [29].

The IB/FE scheme is implemented in the open-source IBAMR software (https://ibamr.github.io) [30], which is an adaptive and distributed memory parallel implementation of the IB method leveraging several open-source computational frameworks, including SAMRAI (https://computation.llnl.gov/projects/samrai) [31], PETSc (https://petsc.org/) [32], libMesh (http://libmesh.github.io) [33] and hypre (http://www.llnl.gov/casc/hypre) to perform core functionality. The simulation is conducted on an Intel® Core™ I9-9980XE CPU at 3.00 GHz machine with 8 cores.

## 3 Results

Results of the flow rate from the 2nd and 3rd cardiac cycles are less than 5% and thus regarded as convergent and stable from the 2nd cycle onwards. The results in the 3rd cycle are presented for demonstration.

Four phases(opening, equilibrium, closing and closed phases) in a venous valve cycle can be observed, each occupying 15%, 30%, 20%, and 35% of the whole cycle time. Fig. 4 depicts 3D morphological changes of valves in four phases from both top and cross-sectional views. When the valves open, the two valve cusps form a mouth-shaped orifice. Throughout the entire cycle in our 3D model, the pair of valves functions more like a membrane, exhibiting a distinct upward bulge during opening and the ability to be squeezed downward during closure. Fig. 5 demonstrates the changes in the magnitude of venous wall displacement over the four phases, with the maximal displacement concentrated in the venous sinus indicating a sinus expansion.

Fig. 6 plots the change of the geometric orifice area(GOA) over time. GOA is crucial for assessing valve performance and blood flow dynamics. It is defined as the cross-sectional area of an elliptical orifice, calculated based on the dimensions of the major and minor axes, assuming an ideal scenario without accounting for flow effects such as vena contracta or pressure losses. It can be observed that the venous valve opens rapidly during the opening phase, then enters the equilibrium period, reaching the maximum opening area of $0.108\;{\mathrm{cm}}^{2}$ at the maximum pressure difference, and closes rapidly during the closing phase, ending in a completely closed state.

**Fig. 7:**
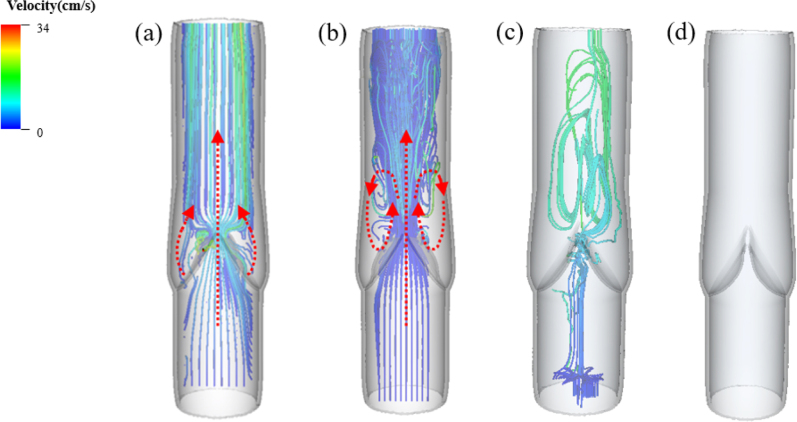
Streamlines of the blood flow in (a) opening, (b) equilibrium, (c) closing and (d) closed phases.

The streamline patterns during the four phases, as shown in Fig. 7, all have their distinct characteristics. During the opening phase, the blood shoots through the narrow channel formed by the two cusps and soon divides into three streams, with the middle stream forming a jet flowing towards the proximal end (Fig. 7(a)). The two side streams begin to return after touching the venous wall and flow towards the venous sinus, forming two vortices during the equilibrium phase (Fig. 7(b)). When the venous valve is about to close, the jet mixes with the side streams and forms an ascending spiral flow (Fig. 7(c)). It is also observed that the vortices in the venous sinus have a strong correlation with the valve motion. Only when the valve closes completely do the vortices vanish (Fig. 7(d)). The velocity in the closed phase appears to suggest no fluid motion because it is minimal compared to the other three phases. Due to the elasticity of the vessel wall and the inertia of the fluid, there is some minor deformation in the wall and sinus, as shown in Fig. 5(d). However, this deformation is small relative to the other three phases.

**Fig. 8:**
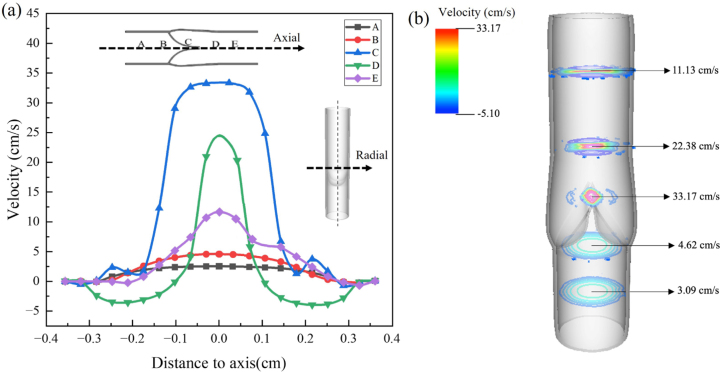
(a) Blood total velocity of five typical points (A: near the distal end; B: approaching the valve; C: between two cusps; D: away from the valve; E: near the proximal end) along the axial direction at the equilibrium phase together with (b) their cross-sectional velocity distributions along the vein.

Five typical points on the central axis of the vein are selected to measure the distribution of the flow velocity along the radial direction at the equilibrium phase. As depicted in Fig. 8(a), the curves are all close to parabolic, indicating layered flow along the vein. Fig. 8(b) illustrates the blood flow velocity along the axial direction at the equilibrium phase, showing that the blood flow accelerates significantly while passing through the cusp “channel” at point C. In particular, the maximum velocity of blood passing through point B is 4.62cm/s, while the transvalvular velocity at point C reaches its maximum at 33.17cm/s. Moreover, there exhibit some minor fluctuations near the sinus wall at point C due to the vortices generated outside the free edges of valve cusps. The minor left–right asymmetries observed in Fig. 7, Fig. 8 could be primarily attributable to the numerical algorithm. The structural finite element mesh and the adaptive fluid mesh are not guaranteed to maintain symmetry throughout the entire simulation. Additionally, minor oscillations of the cusps appear during the equilibrium phases, likely caused by the presence of vortices. More notably, during the closing phase, the fluid domain becomes disturbed, exhibiting a helical flow pattern. The robustness and the accuracy of the immersed boundary method with finite element elasticity (IB/FE) have been investigated with details in the previous work [18], [34], [35].

**Fig. 9:**
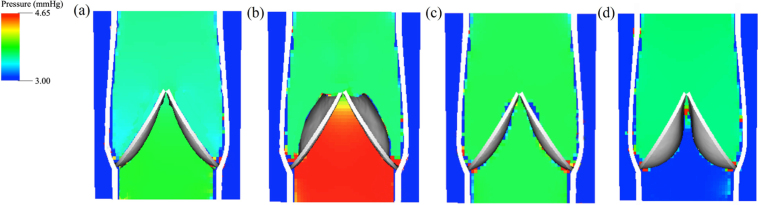
Fluid pressure in (a) opening, (b) equilibrium, (c) closing, and (d) closed phases.

Blood pressure is a crucial indicator of blood circulation function and is widely utilised in clinical examinations. The blood pressures during the four phases are depicted in Fig. 9. The valve opens at a driving pressure difference of around 0.075mmHg and reaches its maximum of approximately 0.75mmHg in the equilibrium phase. Consequently, the venous valve effectively reduces the pressure from the distal side to protect the venous tissue at the proximal side. As the venous valve closes, the pressure on both sides becomes more equal. Once closed, blood begins to exert pressure from the proximal side due to the effect of gravity, and venous valves withstand significant transvalvular pressure, preventing blood from passing through the valve.

**Fig. 10:**
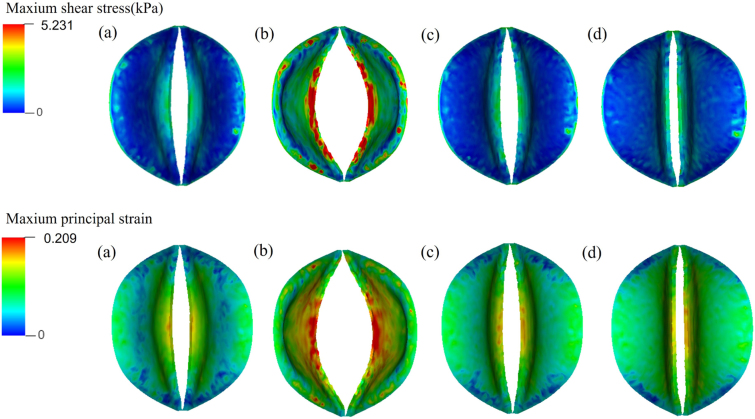
Maximum shear stress and maximum principal strain distributions in (a) opening, (b) equilibrium, (c) closing, and (d) closed phases.

Understanding stress and strain distribution in venous valves helps indicate the occurrence of venous diseases and informs the design of prosthetic valves. Fig. 10 demonstrates the distribution of maximum shear stress $\tau_{max}$ on the venous valve. According to Tresca’s criterion, also known as the Maximum Shear Stress Criterion, $\tau_{max}$ is calculated as half of the difference between the maximum and minimum principal stresses: $\tau_{max}=\left(\sigma_{1}-\sigma_{3}\right)/2$, where $\sigma_{1},\sigma_{3}$ are the maximum and minimum principal stresses. The maximum shear stress increases with the degree of valve opening and is concentrated at the free edge of the venous valve, suggesting that these areas are more susceptible to venous valve disease. The distribution of the maximum principal strain is similar to that of the maximum shear stress, as shown in Fig. 10.

## 4 Discussion

The study highlights the dynamics of valve motion and blood flow patterns across the four phases, aligning well with established physiological phenomena [36]. The flow velocity along the vein’s radial direction is consistent with findings by Helps et al. [37] regarding blood flow characteristics in veins. As shown in Fig. 8(b), the observed maximal transvalvular velocity of 33.17cm/s closely resembles the velocity of 40cm/s reported in the 2D numerical research in [13] with same dimensional parameters adopted from a typical bovine saphenous vein. The velocity also falls within the range of 20.7±18.3cm/s reported in human lower extremity veins [38]. It is acknowledged that the diameter of a bovine saphenous vein typically ranges from 0.5 to 1 cm [13], [39]. In this study, the diameter used is 0.691 cm. For human saphenous veins, the diameter of veins can vary significantly, generally ranging from 0.3 to 1 cm [14], [23], [40]. This variation can be influenced by factors such as age, sex, and individual anatomy. Therefore, bovine veins are commonly used to provide a readily available and cost-effective alternative to human tissues while offering valuable insights applicable to human physiology.

Our 3D modelling highlights a significant disparity between the motion of valves depicted in Fig. 4 and studies utilising 2D modelling [13], [14], [41]. In 2D models, valves are akin to cantilever beams, especially in their opening configuration, where they can open widely, even bending towards the sinus wall. The maximal shear stress and maximal principal strain tend to concentrate at the roots of the 2D valve, contrasting with our 3D valves, which concentrate at the free edges, as illustrated in Fig. 10. This discrepancy may be attributed to the fact that the commissures of the two cusps join together with the sinus wall which significantly restricts valve opening and cannot be adequately captured by a 2D model. Consequently, our model yields a smaller maximal geometric orifice area (GOA) of $0.108\;{\mathrm{cm}}^{2}$ compared to the $0.40\;{\mathrm{cm}}^{2}$ obtained in 2D modelling [13]. When the valve opening reaches its maximum, the length of the short axis of the GOA measures 0.19 cm, while the diameter of the venous inlet end is 0.70 cm. Their ratio, at 27.6%, closely approximates the observed ratio of 37±6% reported in the great saphenous vein of the lower extremities [36].

**Fig. 11:**
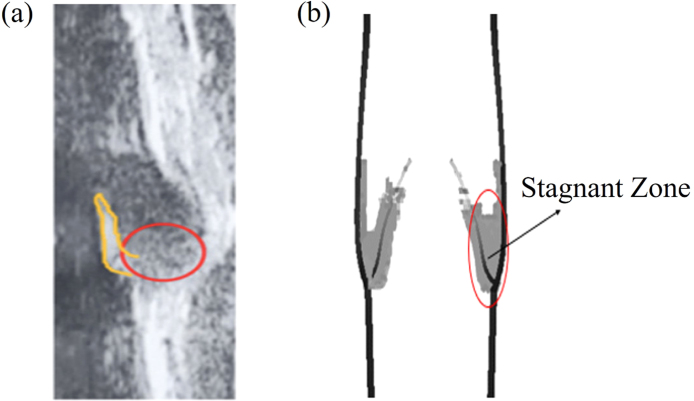
The blood stagnant zones highlighted by red circles (a) in an ultrasound image and (b) in our simulation in the equilibrium phase.

The blood stagnant zone in the vein is a valuable clinical indicator, typically displaying as a grey region on venous ultrasound images (highlighted between the venous valve and sinus in Fig. 11(a)). Blood flow velocity in this zone is typically slow, which significantly increases the risk of blood clot formation. It is widely recognised that blood flow velocity in the stagnant zone is less than 1cm/s[42]. Fig. 11(b) then illustrates the simulated blood stagnant area in the equilibrium phase, with shaded regions indicating where the blood flow velocity throughout the venous sinus is lower than 1cm/s, which correlates well with observations in ultrasound images. A near-stasis region increases the likelihood of thrombus formation in susceptible individuals. If a blood clot (often originating from a deep vein thrombosis) were to form and break loose, travel through the bloodstream, and lodge in the pulmonary arteries in the lungs, it could lead to serious complications known as pulmonary embolism [43].

**Fig. 12:**
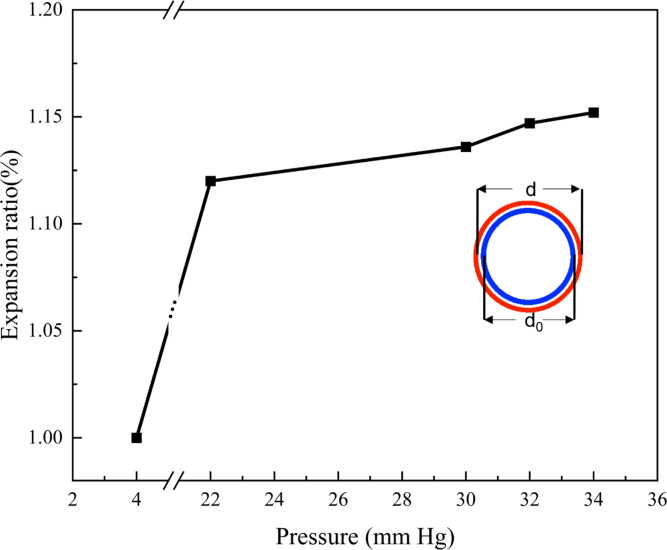
The venous wall expansion ratio $d/d_{0}$ changes with the outlet pressure at the proximal end.

To investigate the impact of blood pressure, considering that blood pressure exceeding 30mmHg is strongly associated with venous ulcer development [44], the hydrostatic pressure at both ends is increased above 30mmHg while keeping the pressure difference constant. It is observed that the changes in velocity and flow rate are not significant. However, there is a noticeable dilation in the venous wall, potentially leading to venous dysfunction. Fig. 12 illustrates the extent of venous dilation, represented by the ratio of the dilated venous diameter d to the reference diameter $d_{0}$. The vein expands by approximately 15% under pressures exceeding 30mmHg.

**Fig. 13:**
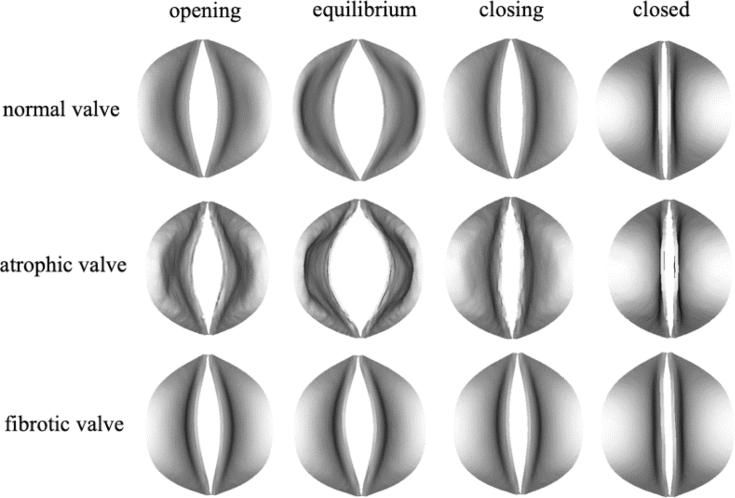
The motion of atrophic and fibrotic valves in four phases compared with the normal valve.

**Fig. 14:**
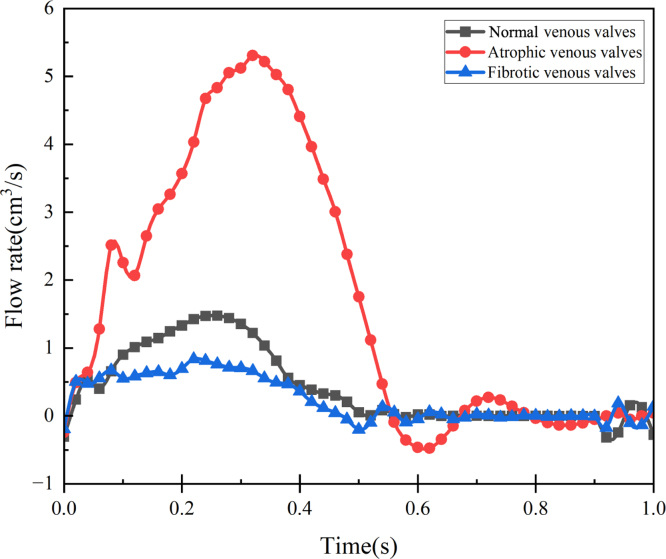
The transvalvular flow rates of normal, atrophic and fibrotic venous valves.

To further investigate the dynamic mechanism of diseased valves, fibrotic and atrophic venous valves are modelled. The pathologies of fibrotic and atrophic valves are simulated by respectively increasing and decreasing the leaflets’ elastic modulus by ten times [45]. The morphology of fibrotic and atrophic venous valves across four phases is depicted in Fig. 13. In cases where the valves are fibrotic, it is difficult for them to open and the free edges struggle to move, resulting in a pronounced bending. Conversely, atrophic venous valves exhibit greater ease of deformation. Fig. 14 illustrates the transvalvular flow rate for all three types of valves. In comparison to normal valves, the blood flow rate significantly increases for atrophic valves, with a delayed maximal flow rate and reflux occurring during closing. On the other hand, the blood flow rate significantly decreases for fibrotic valves, suggesting a heightened propensity for venous thrombosis. The numerical data quantifies the extent of this flow reduction, offering insights valuable for both clinical applications and the design of artificial venous valves.

The study presented here delves into the feasibility of FSI modelling of venous valves. To enhance and further extend the model, sequential pairs of valves and bifurcated venous veins can be modelled to explore the cooperation between adjacent valves. Additionally, the anisotropic fibre contribution to the material property is not considered in the model, and this simplification facilitates a comprehensive comparison with the existing 2D research [13]. It is also acknowledged that the idealised model may not fully capture all anatomical details needed for immediate surgical application, it serves as a valuable stepping stone towards more anatomically accurate and personalised simulations.

Simple pressure conditions are used in this study to maintain computational efficiency and focus to demonstrate the feasibility of constructing a 3D framework. However, more complex boundary conditions, such as Windkessel models, which simulate the compliance and resistance of the vascular system, would provide a more physiologically accurate representation. If more detailed data is provided, implementing these models could enhance the overall accuracy of simulations, especially for future applications involving more specific studies of venous hemodynamics. This underscores the importance of studying how pathological conditions – such as softer or stiffer valve leaflets – affect both the size and persistence of the near-stasis region, as depicted in Fig. 11. Investigating these relationships in future works could yield valuable insights into the risk factors for thrombus formation and subsequent pulmonary embolism, ultimately guiding more effective preventative measures. Future studies will also incorporate the periodic muscular forces around the venous wall resulting from the contraction of surrounding muscles.

Although 2D simulations can be informative, they fall short in capturing the complex three-dimensional dynamics of valve leaflets and the fluid–structure interactions that occur in real systems. This is precisely why we have developed a 3D model for venous valve simulation. Our 3D approach allows us to accurately represent the geometric and functional characteristics of the valve, including the complete range of leaflet movements and interactions with the surrounding venous walls. As demonstrated in Fig. 10, the stress distribution across valve leaflets in our 3D model differs significantly from that in 2D models, highlighting the limitations of 2D simulations in capturing realistic opening configurations.

While this study primarily focuses on the numerical modelling of venous hemodynamics, particularly in a fully 3D framework, the findings have important implications for clinical practice, especially in the diagnosis, treatment, and prevention of venous diseases. The numerical analysis of venous valve motion, especially in fibrotic and atrophic conditions, is highly relevant to understanding how valve dysfunction contributes to venous stasis, which is a key factor in the development of chronic venous insufficiency (CVI) and deep vein thrombosis (DVT) [1], [2]. By quantifying stress–strain distributions and blood flow characteristics, our study offers insights into how valve pathology can lead to disease progression. The results demonstrate that fibrotic valves significantly reduce blood flow rates, increasing the risk of venous thrombosis. This finding could aid clinicians in identifying patients with early-stage fibrosis, potentially guiding earlier intervention strategies. Furthermore, the 3D fluid–structure interaction (FSI) framework developed in this study can be a valuable reference for the design and testing of artificial venous valves. Understanding the stress concentrations and flow patterns can lead to improvements in the durability and function of these devices, which will ultimately benefit patients undergoing valve replacement therapies. In terms of medical impact, the model has strong potential to guide surgical interventions and preventive measures. The data on venous valve mechanics reveal regions of high stress, such as the free edges of the valve leaflets, which are prone to wear and tear. This information can be crucial when assessing the feasibility of valve repair or replacement in a clinical setting. The increased hydrostatic venous pressure studied in the simulations and its impact on vessel dilation can also offer a mechanistic basis for early detection and preventive strategies in patients at risk of venous insufficiency. Our results may help identify patients who could benefit from compression therapy or pharmacological interventions. Additionally, by comparing the effects of fibrosis and atrophy on venous valve function, the study helps delineate the distinct pathological impacts of these conditions, which could assist in developing targeted therapeutic approaches.

## 5 Conclusion

We have developed a fully 3D computational model for coupling the motion of intravenous blood and venous structure, particularly focusing on venous valves. This study integrates FSI with hyperelastic constitutive modelling and typical human venous sizes. Our model accurately captures the periodic valve motion and blood flow patterns across four phases: opening, equilibrium, closing and closed phases. Quantitative factors, including geometric orifice area, flow velocity, flow rate, and the presence of blood stagnant zones, are investigated. The results display the dynamic movement of venous valves and the distribution of stress and strain, revealing significant differences and opposite concentrations compared to 2D venous valve models. This underscores the necessity of 3D modelling in capturing the complexity of venous valve dynamics. Both normal control and pathological conditions, specifically focusing on fibrosis and atrophy of the valves, are explored. Our findings suggest that fibrotic valves are more likely to lead to venous thrombosis, while atrophic valves are prone to blood reflux. Overall, our coupled model aligns well with clinical observations and offers valuable insights into understanding the mechanisms of venous disease and informing the design of prosthetic valves.

## CRediT authorship contribution statement

**Bo Wang:** Writing – original draft, Visualization, Validation, Methodology, Investigation, Formal analysis. **Liuyang Feng:** Writing – review & editing, Resources, Methodology. **Lei Xu:** Writing – review & editing, Visualization, Investigation. **Hao Gao:** Writing – review & editing, Resources, Investigation, Funding acquisition. **Xiaoyu Luo:** Writing – review & editing, Validation, Investigation. **Nan Qi:** Writing – review & editing, Supervision, Resources, Project administration, Methodology, Funding acquisition, Formal analysis.

## Declaration of competing interest

The authors declare that they have no known competing financial interests or personal relationships that could have appeared to influence the work reported in this paper.
